# Role of IgM Memory B Cells and Spleen Function in COVID-19

**DOI:** 10.3389/fimmu.2022.889876

**Published:** 2022-06-30

**Authors:** Carlo Maria Rossi, Marco Vincenzo Lenti, Stefania Merli, Antonio Di Sabatino

**Affiliations:** University of Pavia, First Department of Internal Medicine, IRCCS San Matteo Hospital Foundation, Pavia, Italy

**Keywords:** plasma cells, hyposplenism, SARS-CoV-2, B cell, IgM memory B cell

## Abstract

IgM memory B cells, are a peculiar subset of memory B cells, which probably originates in the spleen and outside germinal centers and provide a rapid line of defence against mucosal infections. Their role in counteracting COVID-19 is still elusive but, recent evidence, mainly boosted by studies on spleen function/involvement in COVID-19, seems to support the notion that this subset of memory B cells could exert a protective role against this virus, along with other coronaviruses, particularly in the acute setting of the infection, as outlined by worst clinical outcomes observed in unvaccinated patients with impaired IgM B memory response and spleen function. Herein we critically summarise the current landscape of studies on IgM memory B cells, focusing on the clinical impact of their depletion, by comparing the COVID-19-related splenic dysfunction with other hypo- and asplenic conditions and by adding recent data on follow-up studies and postulate a mechanistic explanation for their reduced numbers. The early detection of an impaired IgM memory B cell response in patients with COVID-19 may contribute to their improved care through different strategies, such as through tailored vaccine strategies, prompt hospital admission and/or administration of anti-infective treatments, thus resulting in an better prognosis, although at present management algorithms are still unavailable. Moreover, further studies with longer follow-up are needed to assess the evolution of COVID-19-associated/exacerbated immune deficit.

## Introduction

Severe acute respiratory syndrome coronavirus 2 (SARS-CoV-2) is the cause of coronavirus disease 2019 (COVID-19) pandemic, which has dramatically impacted our globalized society, with more than 500 million reported infections and more than6million deaths worldwide as of April 2022 (1). SARS-CoV-2 has been shown to elicit a strong immune activation mirrored by the so-called “cytokine storm” and the complex interaction between the virus and immune system contributes shaping the heterogenous landscape of COVID-19-related pathology, including lung, liver, skin and spleen or other lymphoid organ damage, among others, and of clinical manifestations (2, 3).

Clinical presentation and outcomes of COVID-19 are highly variable, including asymptomatic, mild and severe cases with lung and/or multiorgan failure, the so-called viral sepsis, and complete resolution without sequelae or death (2). Symptoms related to COVID-19 may also be persisting, defining the so-called long COVID syndrome (4). Yet immunological correlates underpinning these disease states have been recently outlined. To control the pandemic, it is vital to characterize the immune response against the virus, and possibly manipulate it, through immunological therapeutical strategies, including among others, vaccines and monoclonal antibodies (5, 6).

An effective antiviral immune response usually requires the coordinated and dynamic interplay of both humoral and cellular effectors, with the participation of both the innate and adaptive arm of immunity, to arrest the spreading of a virus, minimize disease severity and prevent reinfection with the same virus strains and, possibly, its variants (7).

Although the precise protective mechanisms against SARS-CoV-2 are still elusive, several features of the immune responses against SARS-CoV-2 have been identified. More precisely, the role of innate immunity and of T cell, both CD4 and CD8, has been recognized as important, particularly for primary infection (8). B cells and antibody-mediated immunity have also been shown to play a prominent role against SARS-CoV-2, since a rapid appearance of virus-specific antibodies is observed in most individuals, and a high titre of neutralizing antibodies to the spike protein and its receptor binding domain (RBD) have been found to confer protection both in humans and animal models (9). Moreover, other antiviral activities of antibodies, including fraction constant (Fc)-effector related-functions, such as antigen-dependent cell-mediated cytotoxicity (ADCC), are thought to play a role (3). In parallel, a compromised humoral development with attenuated IgG responses has been found to be associated with worse outcomes in acute patients with moderate-to-severe disease (5). In addition, B cell deficiency states, including X-linked agammaglobulinemia (XLA), Good’s syndrome, and common variable immunodeficiency (CVID), at least in some patients, or following monoclonal antibody therapy, seem to be associated with a worse prognosis according to some studies (9). Moreover, the possible beneficial effect in the acute setting of the convalescent plasma therapy, as shown by systematic reviews and meta-analyses, in terms of reduced mortality, increased virus clearance and clinical improvements, may point at a possible role of the antibody response in counteracting the infection, at least in more severe cases (6, 10).

However, the role of antibody-immunity remains a matter of debate, principally due to some inconsistencies (11). In some studies, patients with CVID, CVID-like disorders or other primary antibody deficiencies were not found to be at increased risk for severe outcomes, or the increased risk only applied to specific subsets of patients, such as those with chronic lung involvement of CVID (12, 13). Moreover, the benefit of convalescent plasma was negligible in non-severe cases and the evidence to recommend its use in severely-ill patients is still, overall, inadequate according to the WHO (14).

Moreover, virus specific antibody titre to the spike, the RBD and the nucleocapsid has been shown to be very heterogenous and to decline over time (15, 16). In spite of waning virus specific antibody titre, antigen-specific memory B cell responses appear to be stable (17).

Given the relatively slow course of the disease in severe and even fatal cases of COVID-19, which have a median disease duration of 22.2 ± 3.6 days (16) the role of the memory compartment, which requires some weeks to assure recall cellular and antibody responses, is deemed particularly relevant. Moreover, to guarantee a long-lasting protective effect, to the same virus strain or different variants, memory compartments exert a pivotal role, as derived from the evidence regarding the immune response to SARS-CoV-2 related viruses, SARS-CoV, and Middle East respiratory syndrome coronavirus (MERS-CoV) (18, 19).

Initially overlooked, memory B cell responses to SARS-CoV-2 have been recently intensively studied, as attested by the rapidly increasing number of published papers, including more than 300 entries as of April 2022, aimed at characterizing their functional landscape during the disease and/or after anti-SARS-CoV-2 vaccines.

Reasons accounting for this renewed interest in memory B cells are tightly linked to the studies on the spleen involvement in COVID-19. Earlier in the epidemic, asplenia was found to confer a mortality risk comparable to other recognised risk factors, such as cardiovascular ones (20). SARS-CoV-2, like other coronaviruses, was shown to display a particular tropism for the spleen, particularly the white pulp, possibly mediated by the angiotensin converting enzyme (ACE)-2 receptor. Moreover, in autopsy studies, white pulp atrophy with reduction/absence of lymphoid follicles was revealed (21). Spleen functional alterations were thus thought to contribute, along with other mechanisms, to the B and T cell lymphopenia which is a typical feature of COVID-19. In addition, given that the spleen marginal zone is the specific site where IgM memory B cells are produced and stored and which exert important protective functions against disseminated infection sustained by encapsulated bacterial and viral infections (such as influenza and HIV), a compromised spleen function was thought to contribute to impaired memory B cell responses.

Concomitantly, it is of importance to identify predisposing conditions associated with impaired generation of memory B cell responses, among them splenic hypofunction state, since this subset of patients may be susceptible to more severe manifestations and/or adverse outcomes and hence may benefit from therapeutical strategies aimed at modifying the immune response against the virus.

We herein summarize in a narrative fashion the existing evidence on the role of IgM B memory cell populations and spleen immune function in COVID-19 and their relationship with disease severity and outcomes, especially in the acute setting. We will also consider the COVID-19-induced spleen dysfunction and draw a parallelism with other hypo- and asplenic states, namely, CVID and splenectomy.

## The Germinal Centre and Extrafollicular Memory B Cell Responses to SARS-CoV-2

Within the B cell follicle, mainly in lymph nodes and the spleen, but also in mucosal tissues, antigen-activated B cells with T cell help may determine the germinal centre (GC) reaction, which is vital for the development of affinity-matured plasma cells and long-lived memory B cells that are collectively responsible for a long term and broad protective immunity (22). Within the GC antibodies with enhanced neutralizing activity and breadth resulting from somatic hypermutations arise through a continuous process of clonal evolution of B cells.

However, besides GC reactions, extrafollicular and/or T cell-independent responses may arise. Their aim is to elicit rapid responses, through the generation of memory B cells and short-lived plasma cells with the production of antibodies with a low level of specificity and thus broad reactivity to different bacteria and viruses. These responses take part in early inflammatory responses and have a role in life-threatening, rapid-developing infections.

Interestingly, the severity of COVID-19 seems to have an influence on the quality of the B cell response, with severe disease being associated with extrafollicular responses and defective GC reactions, correlating with high levels of pro-inflammatory cytokines and reduced T follicular helper numbers (19, 23). Memory B cells in this instance display a low level of somatic hypermutation. Whereas in mild disease, both GC- and extrafollicular reactions arise, in which naïve and seasonal coronavirus-specific memory B cells differentiate into activated B cells and short-live plasma cells (24).

**Figure 1:**
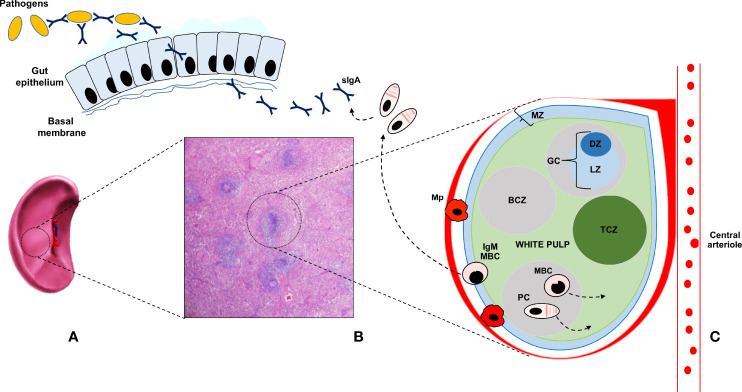
The human splenic is depicted in **(A)**; whereas its histologic appearance is shown in **(B)** The main part of the splenic tissue is the red pulp, whereas the white pulp represents less than 25 percent of the volume. Antigens reach the spleen only through the blood stream, *via* the splenic artery (not depicted for clarity), since the spleen lacks lymphatic vessels. The white pulp is made up of multiple lymph node-like regions, which are embedded in the red pulp, without a capsule. In **(C)** the ultrastructure of the white pulp is represented. The white pulp is surrounded by a layer of innate cells, like specialized macrophages subsets (Mp), making up the marginal zone (MZ) in mice and the perifollicular zone in humans. Bridges channels connecting the red and white pulp are not shown for clarity. Within the white pulp, different compartments can be identified, such as the B cell zone (BCZ) and the T cell zone (TCZ), which in humans is also called periarteriolar lymphoid sheath (PALS). In the BCL, germinal centers (GC) are found. They comprise a dark zone (DZ) and a light zone (LZ). In the DZ, B cells undergo cell division and somatic hypermutation; in the LZ, B cells with productive rearrangements in their B cell receptor, present antigens to T cells and differentiate into memory B cells (MBC) and plasma cells (PC), during germinal center reactions. MBC display migratory features to the extrafollicular areas of the spleen, as suggested by the arrow, and reach lymph nodes *via* the blood. After antigen encounter, MBC give rise to antibody-secreting PC. MBC can also re-enter the GC to acquire increased affinity for the antigen. PC preferentially migrate to the bone marrow, where they constitutively secrete antibodies with a precise specificity. GC- and T- cell independent reactions are also depicted. IgM memory cells (IgM-MBC) arise outside GC, mainly in the MZ. In humans, IgM-MBC are migratory; their trafficking to the gut epithelium is suggested by means of a dashed arrow. In the gut, IgM MBC give rise to IgA-secreting PC, which mediate mucosal immune responses against bacteria and viruses, through the production and delivery up to the intestinal lumen of secretory IgA (sIgA).

As compared to switched memory B cells, which are generated in GC reactions, IgM^+^ IgD^+/-^ CD27^+^ B lymphocytes, also known as IgM memory B cells, seem to arise from a different lineage and develop through GC- and T- independent reactions **Figure 1** (25, 26). IgM memory B cells, which are also known as innate IgM memory B cells, natural memory, or marginal zone B cells, are found in the spleen and peripheral blood, respond to bacterial polysaccharide antigens, and display functional similarities with mouse B-1a cells, the major source of secretory (natural) IgA antibodies (sIgA) lining the intestinal epithelium. Toll-like receptor 9 stimulation has been shown to induce the *in vitro* generation of human IgM memory B cells from transitional B cells (27). Moreover, it has been recently observed that this subset may differentiate *in vitro*, with a T cell-independent mechanism, into IgA secreting plasma cells in the lamina propria and the lymphoid tissue in the gut (25). SIgA exert an important protective function and constitute one of the most relevant components of the mucosal barrier, binding a vast array of antigens and thus preventing the dissemination of bacteria and the entry of allergens and viruses through the formation of immune complexes with cognate Fc receptors.

Moreover, in immunocompetent patients, who were splenectomised for traumatic causes, a depletion of IgM memory B cells has been observed, together with a marked reduction of intestinal IgA^+^ plasma cells and a long-lasting defect in the IgA lining, as assessed up to 15 years after splenectomy. This deficit was thus not compensated by GC dependent mechanisms in lymph nodes and in the mucosa-associated lymphoid tissue of the gut by naïve or transitional B cells (25). Parallelly, in the mouse model, B-1a cells and sIgA are not detectable in the gut of asplenic mice (28). Moreover, in the subset of CVID patients with a IgM memory B cell deficiency the IgA layer in the gut is absent (25).

This clinical and experimental evidence has led the researchers to hypothesise the existence of a functional spleen-gut axis, which is characterised by the trafficking of IgM memory B cells from the spleen to the gut mucosa, where they undergo class-switch to IgA and coordinate mucosal immune responses (25). Interestingly, this immune response seems to be evolutionary conserved, being present also in fish species, which are devoid of bone marrow, lymph nodes and GCs. In fish, dimeric immunoglobulin, IgT, resembling IgA, are generated in the spleen and transported to the gut (29).

These findings may have relevant clinical implications since SARS-Cov-2 like among other viruses associated with pandemics, such as influenza virus, show a mucosal tropism and elicit mucosal inflammation. Mucosal memory B cell responses could contribute to viral clearance during reinfection through a rapid and local increase in IgA antibody levels and to sterilizing immunity at mucosal surface, thus limiting the spread of variants (13, 30).

Taken together, these observations constitute the rationale for assessing the role of IgM memory B cells in COVID-19, particularly in the primary infection and, possibly, also in reinfections and in asymptomatic carriers.

## The Role of IgM Memory B Cells in COVID-19

Few studies have primarily addressed the role of IgM memory B cells in COVID-19 (**Table 1**).

**Table 1:** Studies primarily evaluating the role of IgM memory B cells in COVID-19.

Author(year)	Country	Type of study	Population	SARS-Cov-2 vaccination	Subset of MBC	Time of evaluation of IgM MBC	Comments
Tian X et al. (31) (2022)	China	Observational	31 convalescent children (0-14 years) with mild COVID-19	NA	-IgM -IgG -IgA	At baseline and at 6-8 months	Higher proportion of recovering patients with IgM^+^ B cells than IgG^+^ IgG+ memory response increases with age as opposed to IgM^+^ and IgA^+^
Newell K et al. (15) (2021)	U.S.	Observational	40 COVID-19 non-hospitalized patients at baseline 15 also sampled at 3 months 24 healthy subjects	NA	-CD27^+^, IgD^+^, -CD27^+^ switched memory -CD24^-^	-69 days after symptoms onset -3 months	IgM memory B cells correlate with virus specific antibodies (IgG1 and IgM) and are stable at 3 months
Mazzoni A et al. (32) (2021)	Italy	Experimental	22 individuals; of which 11 with previous infection	mRNA (II doses)	-CD27^+^ IgM^+^ memory -CD27^+^ IgA -CD27^+^ IgG	7, 14, 21, 28 days	IgM^+^ increase only after II dose in COVID-19-naïve individuals, while they increase after I dose in those who had COVID-19
Anand S et al. (33) (2021)	Canada	Observational	32 convalescent individuals sampled up to 31 weeks (n=13)	NA	-IgM	6,11,21,31 weeks	IgM^+^ decrease over time while IgG^+^ are stable
Yang J et al. (34) (2021)	China	Observational	55 recovered patients, 55 healthy donors	NA	-IgM^+^ -IgM^-^	On average 42.2 days after discharge	Lower frequency of IgM isotype-switched memory B cells in recovered patients as compared to heathly donors
Piepenbrink M et al. (35) (2021)	U.S.	Experimental	Hamsters	NA	IgM MBC	Acute infection	Intraperitoneal/aerosol delivery of a human mAb derived from a COVID19 RBP specific IgM memory B cell reduces respiratory tract viral burden/pathology
Lenti MV et al. (36) (2020)	Italy	Observational	63 patients, 3 splenectomised patients excluded	NA	IgM^+^ IgD^+^, CD27+	Acute infection, median 25 days	IgM MBC depletion in 87% of patients
De Biasi S et al. (37) (2020)	Italy	Observational	14 hospitalized patients with pneumonia 11 healthy subjects as controls	NA	-IgM MBC -switched -unswitched	Acute infection, 2-4 days following admission	Decreased number of MBC

MAb, monoclonal antibody; MBC, memory B cell, NA, not assessed.

They vary in terms of the studied population -paediatric or adult-, the setting of disease, -acute leading to hospitalisation or not- or convalescent, and to various memory B cell subsets, together with different times of evaluation. Almost all studies refer to unvaccinated patients. One limitation of most of these studies refers to the age of the heathy controls used for comparison. These are usually much younger than the hospitalised patients, with multimorbidity. This may constitute a bias since memory B cell numbers are known to be reduced with aging (38).

In a US study evaluating memory B cell subsets, in recovered non-hospitalized patients with COVID-19, a negative correlation between the duration of symptoms and frequency of memory B cells, including the IgM subset, was found (15). The likelihood that this result was due to a sample time bias was ruled out by the relatively stable number of memory B cells. Of note, a correlation between total B memory cells with RBD antibodies, both IgG1 and IgM, was found. Interestingly, by analysing memory B cell subsets, this correlation was only present in the IgM^+^ one. To explain this paradoxical finding, since IgM memory B cells do not usually produce switched immunoglobulins, the Authors postulate that a subset of IgM memory B cells could have undergone class switching to IgG with or without entering a germinal center, possibly following a related-coronavirus infection or have originated from T-cell independent pathways, possibly from circulating marginal zone- like cells, given their low CD38 expression.

In a study from our centre located in Northern Italy, close to the first COVID-19 outbreak, and enrolling 66 patients admitted to an Internal Medicine ward, 87.3% of them were found to have IgM memory B cell depletion (defined as absolute counts of IgM^+^ IgD^+^ CD27^+^ <26/microliter) as compared to 25 healthy volunteers. Splenectomy was an exclusion criterium for enrolment. Around 28% of patients died during the hospitalisation. Of note, all patients with adverse outcome were IgM memory B cell deficient and had an intervening infection. Interestingly, IgM memory B cell depletion had an independent prognostic effect on mortality, in the absence of other statistically associated prognostic factors, such as male sex, age, multimorbidity, and total peripheral lymphocyte depletion (36). Additionally, in a Chinese study enrolling hospitalised paediatric patients with mild COVID-19, a strong virus-specific IgM memory B cell response was observed regardless of age (31).

Taken together, these observations seem to associate IgM memory B cell changes with different COVID-19 severity states and outcomes. More precisely, a reduction of this subset correlates with more severe presentations and unfavourable outcomes, whereas a robust IgM memory B cell response is present in patients with a milder or more rapidly resolving forms of the disease.

To corroborate these clinical findings, the IgM memory B cell-mediated response has found to be beneficial in a hamster model of COVID-19. More precisely, 1212C2, a functional antibody derived from a IgM memory B cell line derived from a COVID-19 patient, was shown to exert a protective and preventive effect when administered intraperitoneally and through aerosolization (35).

Surprisingly, the analysis of RBD-specific IgA in COVID-19 patients has reserved little attention despite their likely protective role in the early phases of the viral infection. The following observations have been made in this regard. A cross-reactive human antibody against SARS-CoV and SARS-CoV-2 was found to have neutralizing properties against SARS-CoV-2 when converted to sIgA (39). Also, virus-specific IgA have been found at low titers in convalescent sera and are stable during an 8-month study follow-up (24). Finally, neutralizing IgA antibodies have also been detected for long periods in the saliva of previously infected patients (40). However, to the best of our knowledge, no study has ever evaluated whether IgM memory B cell-deficiency correlated with the depletion of virus-specific IgA at a mucosal level. All these findings may potentially have a clinical implication for stratifying the risk of severe and disseminated COVID-19, even though the translational application in humans still need to be ascertained (2).

The knowledge of the natural evolution of the IgM memory B cell response in COVID-19 is still elusive. According to one study, virus specific IgM memory B cell counts appear stable at 3 months (15), whereas at week 31 their numbers are reduced according to another study (33). Additional prospective studies with longer follow-up are warranted to study the kinetic of the alteration in the IgM memory population and its clinical effects.

Moreover, the exact mechanisms underlying this deficit require further research. In addition to the COVID-19-mediated splenic hypofunction, other mechanisms may be implicated. In the acute setting, TNF, IL6 and other mediators of the cytokine storm, have been shown to influence B cell differentiation, activation and survival, leading to a complex B cell compartment alteration, with recruitment of more immature cells, such as transitional cells, as a consequence of mature B cell exhaustion (37).

Interestingly, a significant reduction (both relative and absolute) of CD27^dull^ memory B cells, has been observed in individuals aged 60 and above (41). This population of memory B cells is largely of IgM isotype, arise in a GC- and T cell independent fashion, displays a reduced mutational status and is necessary for the production mucosal sIgA. This population bears great resemblance to the IgM memory B cells subset (25).

Advanced age is one the main recognized factors accounting for the increased mortality rate in hospitalized patients with COVID-19 which may be related to CD27^dull^ memory B cell depletion in this subset of patients. COVID-19 may thus syndemically interact with pre-existing risk factors, such as age-related immune dysfunction, accounting for unfavourable outcomes (42, 43).

Taken together, these observations put forward the possibilities that the reduced IgM memory B cell pool in COVID-19 patients may be related to the COVID-19-related spleen damage and/or to the age-related B memory cell dysfunction, that may be already present in older patients suffering with COVID-19.

## Assessing Spleen Immune Function in COVID-19

Spleen filtering function can be assessed by counting pitted red cells (PRC), i.e., erythrocytes with membrane alterations, the so-called “pits”, which are detectable under interference phase microscopy on peripheral blood samples (44, 45). A functional impairment is present when more than 4% PRC -out of 1000 counted red cells- are detected (46). This is often the case of disorders such as untreated or refractory celiac disease and other gastrointestinal immune-mediated disorders, such as inflammatory bowel disease, which are frequently characterised by functional hyposplenism (47). Given the association between the filtering and immune spleen function, the evaluation of the haemocatheretic ability of the spleen constitute a surrogate for the analysis of the immune function of the spleen (46).

In the aforementioned study comparing the median counts of IgM memory B cells and of PRC in acute COVID-19 patients, as opposed to hyposplenic and asplenic ones, no inverse correlation despite a reduction of memory B cells was found (36). This finding can be explained by the fact that in patients with acute COVID-19 the haemocateretic function of the spleen seems to be preserved and consequently no PRC increase is usually observed as opposed to its immune function, which appears to be precociously impaired, due to the selective virus-induced damage of the white-pulp and marginal zone as attested by the autoptic exam carried out in a subgroup of patient of our cohort (48). Consequently, the quantification of IgM memory B cells in patients with COVID-19 in the acute phase is the sole method for measuring the spleen immune function. At the same time, the long-term effects of COVID-19 on the IgM memory B compartment are not known at present.

## Clinical and Therapeutical Implications of a Dysfunctional IgM Memory B Cell Response

An intact IgM memory B cell response and spleen immune function seems to exert a relevant role in the acute setting of COVID-19 acting, as a first and broadly reactive defence system against SARS-CoV-2, possibly arising from previous contacts with related coronaviruses. At present, it is not known which is the optimal management for patients displaying a derangement in the IgM memory B cell response during or following COVID-19, particularly in terms of vaccination for encapsulated bacteria (49). Accordingly, it can be presumed that their care should be like that of splenectomised patients, but further studies are needed to assess the persistence of the immune deficit in the long run and the infectious risk of these patients. Therefore, prompt hospital admission and/or administration of anti-infective treatments should be advised in case of acute infections. Parallelly, in this subset of patients, vaccinal measures for SARS-CoV-2 appear vital, despite the fact that their efficacy of remains to be ascertained and that tailoring as to the type of vaccines, the timing of administration and the number of booster doses, is probably needed.

The only study available addressing the response to SARS-CoV-2 vaccines in asplenic patients is a cross-sectional study evaluating humoral titers to SARS-Cov-2 spike protein in patients with thalassemia mayor, also including splenectomized patients. Interestingly, splenectomized patients were found to have high titers of antibodies, comparable to healthy individuals. None of the patients was infected by COVID-19 during the 6-month follow-up (50).

## Outlook

Further studies on larger populations and registries are therefore awaited to develop diagnostic and management guidelines for patients with IgM B memory depletion and spleen dysfunction, this latter either associated with COVID-19 or pre-existent. More in depth, it is still undefined how to manage patients with a pre-existing spleen hypofunction, such as patients with CVID and other primary immunodeficiencies or immune- mediated or autoimmune gastrointestinal disorders, namely celiac disease and inflammatory bowel disease (28). It is in fact well known that these patients may be predisposed to the development of severe and invasive infections, such as invasive pneumococcal disease. Nonetheless, real-world evidence on COVID-19 in patients with coeliac disease or inflammatory bowel disease does not seem to point at a more severe viral infection compared to the general population (48, 51). To note, these patients were not stratified according to the presence or absence -and the quantification- of hyposplenism and IgM memory B cell depletion. All these issues should be the focus of future studies.

## Author Contributions

All authors significantly participated in the drafting of the manuscript or critical revision of the manuscript for important intellectual content and provided approval of the final submitted version. Individual contributions are as follows: CR wrote the manuscript, ML and SM reviewed the manuscript. AS reviewed the paper and made final critical revision for important intellectual contents.

## Funding

Italian Ministry of Health. Grant number: Rete Aging-Project PROMISING, RCR-2021-23671216. PI of the project: ML.

## Conflict of Interest

The authors declare that the research was conducted in the absence of any commercial or financial relationships that could be construed as a potential conflict of interest.

## Publisher’s Note

All claims expressed in this article are solely those of the authors and do not necessarily represent those of their affiliated organizations, or those of the publisher, the editors and the reviewers. Any product that may be evaluated in this article, or claim that may be made by its manufacturer, is not guaranteed or endorsed by the publisher.
